# 1-(3-Chloro­pyridin-2-yl)hydrazine

**DOI:** 10.1107/S1600536810036950

**Published:** 2010-09-18

**Authors:** Peng Wang, Rong Wan, Peng Yu, Qiu He, Jian-qiang Zhang

**Affiliations:** a Department of Applied Chemistry, College of Science, Nanjing University of Technology, No. 5 Xinmofan Road, Nanjing, Nanjing 210009, People’s Republic of China

## Abstract

The title compound, C_5_H_6_ClN_3_, was synthesized by the reaction of 2,3-dichloro­pyridine and hydrazine hydrate. An intra­molecular N—H⋯Cl hydrogen bond results in the formation of a planar (mean deviation 0.038 Å) five-membered ring. In the crystal, inter­molecular N—H⋯N hydrogen bonds link the mol­ecules into a three-dimensional network.

## Related literature

The title compound is an inter­mediate in the synthesis of Rynaxypyr, a new insecticidal anthranilic diamide. For the synthesis and biological properties of Rynaxypyr, see: Lahm *et al.* (2007 ▶). For standard bond lengths, see: Allen *et al.* (1987 ▶).
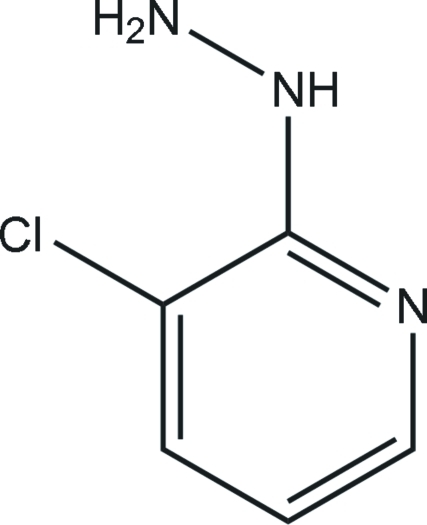


## Experimental

### 

#### Crystal data

C_5_H_6_ClN_3_
*M* *_r_* = 143.58
Monoclinic, 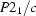

*a* = 11.637 (2) Å
*b* = 3.9060 (8) Å
*c* = 13.946 (3) Å
β = 103.46 (3)°
*V* = 616.5 (2) Å^3^
*Z* = 4
Mo *K*α radiation
μ = 0.52 mm^−1^
*T* = 293 K
0.30 × 0.20 × 0.10 mm

#### Data collection

Enraf–Nonius CAD-4 diffractometer
Absorption correction: ψ scan (North *et al.*, 1968 ▶) *T*_min_ = 0.860, *T*_max_ = 0.950
2173 measured reflections
1124 independent reflections
936 reflections with *I* > 2σ(*I*)
*R*_int_ = 0.036
3 standard reflections every 200 reflections  intensity decay: 1%

#### Refinement

*R*[*F* ^2^ > 2σ(*F* ^2^)] = 0.032
*wR*(*F* ^2^) = 0.082
*S* = 1.04
1124 reflections
91 parameters
H atoms treated by a mixture of independent and constrained refinement
Δρ_max_ = 0.17 e Å^−3^
Δρ_min_ = −0.15 e Å^−3^

### 

Data collection: *CAD-4 EXPRESS* (Enraf–Nonius, 1994 ▶); cell refinement: *CAD-4 EXPRESS*; data reduction: *XCAD4* (Harms & Wocadlo, 1995 ▶); program(s) used to solve structure: *SHELXS97* (Sheldrick, 2008 ▶); program(s) used to refine structure: *SHELXL97* (Sheldrick, 2008 ▶); molecular graphics: *SHELXTL* (Sheldrick, 2008 ▶); software used to prepare material for publication: *SHELXTL*.

## Supplementary Material

Crystal structure: contains datablocks global, I. DOI: 10.1107/S1600536810036950/im2217sup1.cif

Structure factors: contains datablocks I. DOI: 10.1107/S1600536810036950/im2217Isup2.hkl

Additional supplementary materials:  crystallographic information; 3D view; checkCIF report

## Figures and Tables

**Table 1:** Hydrogen-bond geometry (Å, °)

*D*—H⋯*A*	*D*—H	H⋯*A*	*D*⋯*A*	*D*—H⋯*A*
N2—H2*A*⋯Cl	0.88 (2)	2.58 (2)	2.970 (2)	108 (2)
N2—H2*A*⋯N3^i^	0.88 (2)	2.28 (2)	3.058 (3)	148 (2)
N3—H3*A*⋯N1^ii^	0.94 (2)	2.41 (2)	3.243 (3)	148 (2)
N3—H3*B*⋯N2^iii^	0.90 (2)	2.68 (2)	3.492 (3)	151 (2)

Symmetry codes: (i) 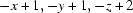; (ii) 
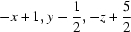
; (iii) 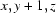.

**Fig. 1.**
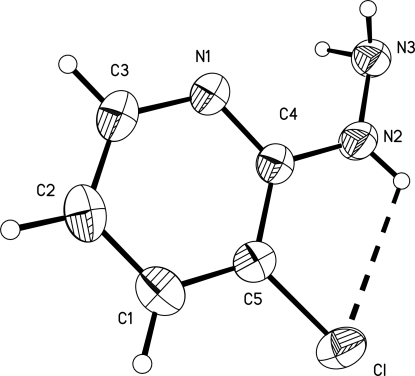
Molecular structure of the title compound with the atom-numbering scheme. Displacement ellipsoids are drawn at the 50% probability level. Hydrogen bond is shown as dashed line.

**Fig. 2.**
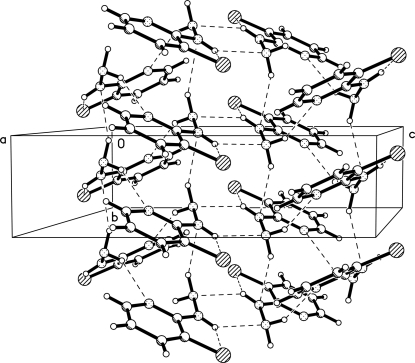
Partial packing diagram of (I). Hydrogen bonds are shown as dashed lines.

## supplementary crystallographic information

### Comment

1-(3-Chloropyridin-2-yl)hydrazine is an important intermediate in the synthesis of Rynaxypyr, a new insecticidal anthranilic diamide, which acts as a potent and selective ryanodine receptor activator. Rynaxypyr is characterized by its high levels of insecticidal activity and low toxicity to mammals attributed to a high selectivity for insect over mammalian ryanodine receptors (Lahm *et al.*, 2007).

We report herein the crystal structure of the title compound,(I). In the molecule of the title compound (Fig. 1), bond lengths (Allen *et al.*, 1987) and angles are within normal ranges. The pyridine ring A(C1/C2/C3/N1/C4/C5) is, of course, planar with a mean deviation from planarity of 0.0027 Å (C1 - 0.0013, C2 - 0.0027, C3 0.0037, N1 - 0.0005, C4 - 0.0034 and C5 0.0042 Å, respectively). An intramolecular N—H···Cl hydrogen bond (Table 1) results in the formation of one planar five-membered ring B(C4/C5/Cl/H2A/N2) with a mean deviation from planarity of 0.0380 Å (C4 0.0119, C5 - 0.0382, Cl 0.0382, H2A -0.0568 and N2 0.0503 Å, respectively). The dihedral angle A/B = 3.5 (1) Å, showing the rings to be almost coplanar. In the crystal structure, three intermolecular N—H···N hydrogen bonds (Table 1) link the molecules to form a three-dimensional network (Fig. 2).

### Experimental

Hydrazine hydrate (10 mmol) was added dropwise to a refluxing solution of 2,3-dichloropyridine (10 mmol) in ethanol. The reaction mixture was stirred and refluxed for 2 h. After cooling and filtering, crude compound (I) was obtained. Pure compound (I) was obtained by recrystallization from THF (15 ml, yield 65%). Crystals of (I) suitable for X-ray diffraction were obtained by slow evaporation of an ethanolic solution.

### Refinement

All H atoms bonded to carbon were placed geometrically with distances of 0.93 Å refined using a riding motion approximation with *U*_iso_(H) = 1.2 *U*_eq_*(C)* of the carrier atom. H atoms at the hyrazido substituent were found in the difference Fourier map and refined freely.

### Crystal data

**Table:**

C_5_H_6_ClN_3_	*F*(000) = 296
*M**_r_* = 143.58	*D*_x_ = 1.547 Mg m^−^^3^
Monoclinic, *P*2_1_/*c*	Melting point = 427–429 K
Hall symbol: -P 2ybc	Mo *K*α radiation, λ = 0.71073 Å
*a* = 11.637 (2) Å	Cell parameters from 25 reflections
*b* = 3.9060 (8) Å	θ = 9–13°
*c* = 13.946 (3) Å	µ = 0.52 mm^−^^1^
β = 103.46 (3)°	*T* = 293 K
*V* = 616.5 (2) Å^3^	Block, yellow
*Z* = 4	0.30 × 0.20 × 0.10 mm

### Data collection

**Table:**

Enraf–Nonius CAD-4 diffractometer	936 reflections with *I* > 2σ(*I*)
Radiation source: fine-focus sealed tube	*R*_int_ = 0.036
graphite	θ_max_ = 25.3°, θ_min_ = 1.8°
ω/2θ scans	*h* = 0→13
Absorption correction: ψ scan (North *et al.*, 1968)	*k* = −4→4
*T*_min_ = 0.860, *T*_max_ = 0.950	*l* = −16→16
2173 measured reflections	3 standard reflections every 200 reflections
1124 independent reflections	intensity decay: 1%

### Refinement

**Table:**

Refinement on *F*^2^	Secondary atom site location: difference Fourier map
Least-squares matrix: full	Hydrogen site location: inferred from neighbouring sites
*R*[*F*^2^ > 2σ(*F*^2^)] = 0.032	H atoms treated by a mixture of independent and constrained refinement
*wR*(*F*^2^) = 0.082	*w* = 1/[σ^2^(*F*_o_^2^) + (0.0422*P*)^2^] where *P* = (*F*_o_^2^ + 2*F*_c_^2^)/3
*S* = 1.04	(Δ/σ)_max_ = 0.001
1124 reflections	Δρ_max_ = 0.17 e Å^−^^3^
91 parameters	Δρ_min_ = −0.15 e Å^−^^3^
0 restraints	Extinction correction: *SHELXL97* (Sheldrick, 2008)
Primary atom site location: structure-invariant direct methods	Extinction coefficient: 0.166 (16)

### Special details

**Table:**

Geometry. All e.s.d.'s (except the e.s.d. in the dihedral angle between two l.s. planes) are estimated using the full covariance matrix. The cell e.s.d.'s are taken into account individually in the estimation of e.s.d.'s in distances, angles and torsion angles; correlations between e.s.d.'s in cell parameters are only used when they are defined by crystal symmetry. An approximate (isotropic) treatment of cell e.s.d.'s is used for estimating e.s.d.'s involving l.s. planes.
Refinement. Refinement of *F*^2^ against ALL reflections. The weighted *R*-factor *wR* and goodness of fit *S* are based on *F*^2^, conventional *R*-factors *R* are based on *F*, with *F* set to zero for negative *F*^2^. The threshold expression of *F*^2^ > σ(*F*^2^) is used only for calculating *R*-factors(gt) *etc*. and is not relevant to the choice of reflections for refinement. *R*-factors based on *F*^2^ are statistically about twice as large as those based on *F*, and *R*- factors based on ALL data will be even larger.

### Fractional atomic coordinates and isotropic or equivalent isotropic displacement parameters (Å^2^)

**Table:**

	*x*	*y*	*z*	*U*_iso_*/*U*_eq_	
Cl	0.18715 (4)	0.20164 (13)	0.95683 (4)	0.0436 (2)	
N1	0.33408 (13)	0.6634 (4)	1.20853 (11)	0.0332 (4)	
N2	0.41097 (14)	0.4864 (5)	1.07771 (12)	0.0394 (4)	
H2A	0.4020 (19)	0.408 (6)	1.0174 (17)	0.059*	
N3	0.51985 (14)	0.6524 (5)	1.11726 (13)	0.0388 (4)	
H3B	0.509 (2)	0.874 (7)	1.1298 (17)	0.058*	
H3A	0.554 (2)	0.586 (6)	1.1821 (16)	0.058*	
C1	0.11354 (17)	0.4002 (5)	1.11712 (14)	0.0374 (5)	
H1	0.0399	0.3118	1.0866	0.045*	
C2	0.13219 (17)	0.5562 (5)	1.21092 (14)	0.0408 (5)	
H2	0.0716	0.5732	1.2439	0.049*	
C3	0.24223 (18)	0.6808 (5)	1.25120 (15)	0.0377 (5)	
H3	0.2545	0.7854	1.3127	0.045*	
C4	0.31800 (15)	0.5159 (4)	1.12028 (13)	0.0286 (4)	
C5	0.20482 (16)	0.3823 (5)	1.07282 (13)	0.0307 (4)	

### Atomic displacement parameters (Å^2^)

**Table:**

	*U*^11^	*U*^22^	*U*^33^	*U*^12^	*U*^13^	*U*^23^
Cl	0.0486 (3)	0.0455 (3)	0.0348 (3)	−0.0082 (2)	0.0057 (2)	−0.0066 (2)
N1	0.0373 (9)	0.0332 (9)	0.0296 (8)	0.0007 (7)	0.0089 (7)	−0.0007 (7)
N2	0.0337 (9)	0.0518 (11)	0.0339 (9)	−0.0069 (8)	0.0103 (7)	−0.0107 (8)
N3	0.0334 (9)	0.0437 (10)	0.0392 (9)	−0.0045 (8)	0.0083 (7)	−0.0041 (8)
C1	0.0358 (10)	0.0331 (11)	0.0436 (11)	−0.0017 (8)	0.0095 (9)	0.0098 (9)
C2	0.0415 (11)	0.0414 (12)	0.0445 (12)	0.0053 (9)	0.0202 (9)	0.0071 (10)
C3	0.0481 (12)	0.0333 (10)	0.0348 (10)	0.0054 (9)	0.0158 (9)	0.0014 (8)
C4	0.0323 (10)	0.0231 (9)	0.0304 (9)	0.0017 (7)	0.0072 (7)	0.0026 (7)
C5	0.0365 (10)	0.0251 (9)	0.0292 (9)	0.0009 (8)	0.0048 (8)	0.0031 (7)

### Geometric parameters (Å, °)

**Table:**

Cl—C5	1.7327 (18)	C1—C5	1.349 (3)
N1—C4	1.332 (2)	C1—C2	1.413 (3)
N1—C3	1.341 (2)	C1—H1	0.9300
N2—C4	1.355 (2)	C2—C3	1.363 (3)
N2—N3	1.416 (2)	C2—H2	0.9300
N2—H2A	0.88 (2)	C3—H3	0.9300
N3—H3B	0.90 (3)	C4—C5	1.428 (2)
N3—H3A	0.94 (2)		
			
C4—N1—C3	118.50 (17)	C3—C2—H2	121.2
C4—N2—N3	121.60 (16)	C1—C2—H2	121.2
C4—N2—H2A	121.3 (15)	N1—C3—C2	124.65 (19)
N3—N2—H2A	115.4 (15)	N1—C3—H3	117.7
N2—N3—H3B	111.6 (15)	C2—C3—H3	117.7
N2—N3—H3A	112.9 (14)	N1—C4—N2	119.14 (16)
H3B—N3—H3A	97.2 (19)	N1—C4—C5	120.15 (16)
C5—C1—C2	118.59 (18)	N2—C4—C5	120.69 (16)
C5—C1—H1	120.7	C1—C5—C4	120.56 (17)
C2—C1—H1	120.7	C1—C5—Cl	120.90 (15)
C3—C2—C1	117.55 (18)	C4—C5—Cl	118.54 (14)
			
C5—C1—C2—C3	0.1 (3)	C2—C1—C5—C4	0.5 (3)
C4—N1—C3—C2	0.3 (3)	C2—C1—C5—Cl	−178.77 (13)
C1—C2—C3—N1	−0.6 (3)	N1—C4—C5—C1	−0.8 (3)
C3—N1—C4—N2	−177.86 (17)	N2—C4—C5—C1	177.42 (18)
C3—N1—C4—C5	0.3 (3)	N1—C4—C5—Cl	178.52 (13)
N3—N2—C4—N1	−9.6 (3)	N2—C4—C5—Cl	−3.3 (2)
N3—N2—C4—C5	172.20 (17)		

### Hydrogen-bond geometry (Å, °)

**Table:**

*D*—H···*A*	*D*—H	H···*A*	*D*···*A*	*D*—H···*A*
N2—H2A···Cl	0.88 (2)	2.58 (2)	2.970 (2)	108 (2)
N2—H2A···N3^i^	0.88 (2)	2.28 (2)	3.058 (3)	148 (2)
N3—H3A···N1^ii^	0.94 (2)	2.41 (2)	3.243 (3)	148 (2)
N3—H3B···N2^iii^	0.90 (2)	2.68 (2)	3.492 (3)	151 (2)

Symmetry codes: (i) −*x*+1, −*y*+1, −*z*+2; (ii) −*x*+1, *y*−1/2, −*z*+5/2; (iii) *x*, *y*+1, *z*.
